# Role of *Lactobacillus rhamnosus* (FloraActive™) 19070-2 and *Lactobacillus reuteri* (FloraActive™) 12246 in Infant Colic: A Randomized Dietary Study

**DOI:** 10.3390/nu10121975

**Published:** 2018-12-13

**Authors:** Sergei Gerasimov, Jesper Gantzel, Nataliia Dementieva, Olha Schevchenko, Orisia Tsitsura, Nadiia Guta, Viktor Bobyk, Vira Kaprus

**Affiliations:** 1 Department of Pediatrics #2, Lviv National Medical University, 69 Pekarska str., 79010 Lviv, Ukraine; 2 BioCare Copenhagen A/S, Ole Maaløes Vej 3, 2200 Copenhagen, Denmark; jg@biocarecph.com; 3 Dnipro State Children Hospital, 13 Kosmichna str., 49100 Dnipro, Ukraine; dementievana@ukr.net (N.D.); oshevchenko2202@ukr.net (O.S.); 4 Ivano-Frankivsk State Children Hospital, 132 Yevhena Konovaltsya str., 76014 Ivano-Frankivsk, Ukraine; vmatsalac@gmail.com; 5 Lviv City Children Hospital, 4 Pylypa Orlyka str., 79059 Lviv, Ukraine; nadiia.guta@gmail.com (N.G.); vbobyk@yahoo.com (V.B.); 6 Lviv Community 4 Clinical Hospital, 3 Yaroslava Stetska str., 79007 Lviv, Ukraine; kaprus4mkl@gmail.com

**Keywords:** infant, colic, lactobacilli

## Abstract

Infant colic is a common condition of unknown pathogenesis that brings frustration to families seeking for effective management. Accumulating evidence suggests that some single strains of lactobacilli may play a positive dietary role in attenuation of colic in exclusively breastfed infants. The objective of this study was to evaluate a mixture of two *Lactobacillus* strains in decreasing infant cry and fuss in this population. Infants aged 4–12 weeks received *L. rhamnosus* 19070-2 and *L. reuteri* 12246 in a daily dose of 250 × 10^6^ CFU, 3.33 mg of fructooligosaccharide, and 200 IU of vitamin D_3_ (84 infants, probiotic group) or just vitamin D_3_ (84 infants, control group) for 28 days. Cry and fuss time were measured with validated Baby’s Day Diary on days 0 and 28. At baseline, mean (SD) duration of cry and fuss time was comparable in the probiotic and control groups: 305 (81) vs. 315 (90) min., respectively (*p* = 0.450). On day 28, mean cry and fuss time became statistically different: 142 (89) vs. 199 (72), respectively (*p* < 0.05). Mean change in cry and fuss time from day 0 through day 28 was −163 (99) minutes in the probiotic and −116 (94) minutes in the control group (*p* = 0.019). Our findings confirm that lactobacilli decrease cry and fuss time and provide a dietary support in exclusively breastfed infants with colic.

## 1. Introduction

Infant colic (IC) represents a temporary self-limited condition, which occurs in about one of five infants within the first months of life, and is characterized by inconsolable cry and fuss of unknown cause [1]. Wessel et al., who coined and brought the term into a wide medical use, defined IC as a stable symptomatic pattern with timing as a rule of three threes in an infant “who is otherwise healthy and well-fed, has paroxysms of irritability, fussy or cry, lasting for a total of three hours a day, occurring on more than three days in any one week for a period of three weeks” [2]. Despite its benign nature, IC serves a significant source for maternal anxiety, and depression [3], impaired family functioning [4], and the most common reason for seeking medical advice in this age group [1]. Infant colic had been linked to poor sleep and disorders in mental health in school age [5]. Due to universal prevalence, economic burden associated with IC is great for the healthcare system. In the UK, annual estimates for financial losses due to IC has been 65 million pounds [6]. At the same time, there is no consensus on the treatment of IC. Most of the current interventions have been found ineffective, unequivocal, or unsafe [7].

There is accumulating evidence that lactobacilli may play a role in IC. Earlier findings showed that infants with IC had more gas-forming *Clostridium difficile*, *Klebsiella pneumoniae*, and *Escherichia coli* in their intestines and lesser microbial diversity than their non-colic counterparts [8,9,10]. Probiotic bacteria can also theoretically influence sulfate reducing bacteria, methanogens and/or acetogens playing an important role in the functioning of the gut [11]. It was hypothesized that inoculation of antagonizing probiotic species into the gut of such infants could potentially decrease gas production, thereby alleviating abdominal distress. However, probiotic supplementation studies are not fully consistent, and their results ranged from significant and meaningful effects through the absence of efficacy [12,13,14,15,16,17]. Meta-analysis of personal data from randomized clinical trials showed that exclusively breastfed infants significantly benefited from probiotic supplementation, while this effect in the formula-fed infant was not evident [18].

The objective of our study was to explore the effect of *Lactobacillus rhamnosus* 19070-2 and *Lactobacillus reuteri* 12246 on the course of colic in the hypothetically susceptible population of exclusively breastfed infants.

The rationale for selecting a combination of *L. rhamnosus* 19070-2 and *L. reuteri* 12246 was based on its safety and clinical efficacy in a series of pediatric studies. Two randomized clinical studies concluded that the combination of the mentioned strains significantly reduced the duration of acute diarrhea [19,20], and results of two other studies reported a reduction in atopic dermatitis relapse rate together with a decrease of associated intestinal permeability, and reduction of gastrointestinal symptoms [21,22]. These studies, which involved 196 children aged six months to thirteen years, suggested that use of *L. rhamnosus* 19070-2 and *L. reuteri* 12246 for five days to six weeks was safe and effective.

## 2. Materials and Methods 

This was a phase II randomized parallel group prospective controlled multi-center dietary study. The case of IC was defined as cry and/or fuss lasting > 3 h a day, occurring > 3 days for the last seven days [23]. Other inclusion criteria were as follows: Informed consent form signed by parents, gender males and females, age on enrollment 4–12 weeks; gestational age 37–42 weeks; birth weight 2500–4200 g, stated availability throughout the study period, stated availability of mobile phone or phone with answering machine. Excluded from the study were infants who received any amount of formula feeding, those who failed to thrive (weight gain less than 100 g per week as averaged from the birth weight to the weight at entry) and those with present intake of antibiotics, prebiotics or probiotics by infant or mother. Also excluded were mothers with current maternal smoking, known moderate or severe disease of any systems (neural, skeletal, muscular, cutaneous, gastrointestinal, respiratory, genital, urinary, immune), difficulty of parents to comprehend study requirements as judged by physician, suspected parental alcohol or drug addiction as judged by physician. There were no changes made to eligibility criteria or other methods after the study commencement.

The study was performed at six (6) clinical centers, where data was collected: Three centers—tertiary care state or city children hospitals (Dnipro State Children Hospital, Dnipro; Ivano-Frankivsk State Children Hospital, Ivano-Frankivsk; Lviv City Children Hospital; Lviv), two centers—out-patient community clinic or out-patient department at hospital (Chernihiv Pediatric Outpatient Clinic #1, Chernihiv; Pediatric Outpatient Clinic at Lviv Community 4 Clinical Hospital, Lviv); one center—University Medical Center (Lviv National Medical University, Lviv). All centers were in Ukraine. Methods of advertisement included social media, verbal information and brochure given during hospital, outpatient visits and regular newborn visits. During newborn visit families were also supplied with a leaflet with study announcement and normal immunization schedule. 

Infants in the probiotic group were administered *L. rhamnosus* 19070-2 and *L. reuteri* 12246 in a dose of 125 × 10^6^ CFU (both strains), 2.5 mcg (100 IU) of vitamin D_3_, and 1.667 mg of fructooligosaccharides (FOS) in sunflower oil drops (0.25 mL). Subjects in the control group received 2.5 mcg (100 IU) of vitamin D_3_ per dose. Both probiotic and control groups were given a dose of the test dietary supplement (TDS) two times daily: One dose (0.25 mL) during the first morning (from 06:00) breastfeeding, and one dose (0.25 mL) during one of the evening (18:00−24:00) breast feedings. As a result, the probiotic group received a daily dose of 250 × 10^6^ CFU of lactobacilli (125 × 10^6^ CFU each strain) with 5 mcg (200 IU) of vitamin D_3_, and 3.33 mg of FOS, while the control group received only 5 mcg (200 IU) of vitamin D_3_. The TDS was dripped behind the gums just before feeding. Compliance was assessed with phone calls (days 12, 26) and 28-Day Study Diary (28-DSD), where caregivers checked morning and evening TDS intakes. The 28-DSD was also designed to screen for mothers’ diet (cow’s milk, eggs, chocolate, nuts), exclusive breast-feeding status, and adverse events that might be linked to infant TDS intolerance (regurgitation, vomiting, constipation, diarrhea, skin rash). To evaluate background conditions that might interfere with the assessment of intolerance events, parents were questioned whether infant ever experienced the above disorders. 

The primary outcome measure was changed in mean cry/fuss time (min/day) from day 0 through day 28. Cry was not specifically defined, while fuss was defined as “behavior that is not quite cry but not awake and content either” [24]. The secondary outcomes included: Time to treatment success, a day when more than or equal to 25% and 50% reduction in cry/fuss time from difference between baseline value and 180 min, a cutoff for definition of IC; recovery success (percent) at 7, 14, 21, 28 days, defined as reduction in duration of cry/fuss time less than 3 h per day (unmet Wessel criteria). For calculation of time to treatment success, data obtained on the days 6–7, 13–14, 20–21, and 27–28, was averaged and means were ascribed to days 7, 14, 21, and 28, respectively. Based on mean values, for every infant we found a day on which cry/fuss time decreased more than 25% or 50% as compared with baseline—180 min value. These days were averaged and mean weekly time to 25% or 50% improvement was calculated for active and control groups. Tertiary outcome measures included: Infant sleep duration (min/day) on days 0, 7, 14, 21, 28; change in maternal depression score from day 0 through 28. There were no changes made to outcomes after the trial commencement.

Cry, fuss, and sleep time were assessed with Baby’s Day Diary (BDD), a validated tool for the observation of infant activity [24]. Briefly, the BDD had four, six-hour time-rulers printed on a single page, used to capture babies’ behaviors on a 24-h basis. Instructions were given verbally by investigators and pre-printed on each BDD. Parents ticked the start and end time of successive periods of behavior on the time rulers, painted these periods with relevant schematic patterns against a scale showing five-minute increments of time. The BDD was completed nine times: On day 0 (before inclusion), and then on pairs of consecutive days 6–7, 13–14, 20–21, 27–28 after the commencement of TDS intake. To improve compliance with BDD recording, parents received two phone call reminders (days 12, 26), and two text messages (SMS) (days 5, 19). During phone calls parents were also asked about their impression of colic (“better”, “worse”, “no changes”). Maternal depression score was estimated with the Edinburgh Postnatal Depression Scale (EPDS) [25]. The scale was linguistically validated. [26]. Formal permissions to use these assessment tools were obtained from copyright holders. 

To detect a difference in change of cry/fuss time of 40 min between the probiotic and control groups on day 28, with power of 80% and confidence of 0.05, 140 infants should be enrolled. At a dropout rate of 20%, the final sample size was 140 × 1.2 = 168. The rationale for the number of infants stemmed from the five published reports on the efficacy of probiotics in IC. It has been estimated weighted mean difference in change of cry and fuss time (40 min) between probiotic and control arms, and population variance (7456) [12,13,14,15,27]. There were no interim analyses and stopping guidelines in this study.

Random numbers were generated by the Random Allocation Software v. 1.0.0 [28]. Randomization was restricted to a 4-block system to secure an equal number of infants in the probiotic and control groups. Random allocation was implemented by sticking the labels with sequential random numbers and sequential selection of packs by the investigator. Random numbers were generated by the technician who did not participate in the distribution of packs among centers or data analysis. Investigators and families were blinded to which supplement was given. The supplements looked the same on appearance, smell and consistency and differed only by the randomization numbers on packs.

Absolute number and percent were used to describe baseline characteristics of boys/girls, proportion of caesarean delivery, family history of atopy, proton pump inhibitor use. Mean and standard deviation described age at study entry (weeks), birth weight (g), gestation (weeks), duration (days) of IC before randomization, total daily cry/fuss time (min/day), fuss time (min/day), cry time (min/day), infant sleep duration (min/day), maternal mental health (EPDS score). Difference between the groups in percent data was assessed in z-test, and between means—in a two-tailed *t*-test. Distribution of data was checked with Kolmogorov-Smirnov test. Recovery from IC was plotted using the Kaplan-Meier method, and the difference in recovery rates was assessed with Cox’s F-test. If not otherwise specified, data in the text is present as mean (SD), and the difference between means is assessed in a two-tailed *t*-test. Tests were performed with Statistica 9 (StatSoft., Inc., Tulsa, OK, US).

The study received positive decisions of Ethical committees at clinical centers (Dnipro State Children Hospital, protocol #6.4 of 29 June 2016; Ivano-Frankivsk State Children Hospital protocol #12 of 6 May 2016; Lviv City Children Hospital protocol #3 of 22 June 2016; Chernihiv Pediatric Outpatient Clinic #1 protocol #11 of 8 September 2016; Pediatric Outpatient Clinic at Lviv Community 4 Clinical Hospital protocol #8 of 21 March 2017; Lviv National Medical University protocol #3 of 14 March 2016). Before enrollment, parents signed an informed consent form. The study protocol was registered by the protocol registration system at Clinicaltrial.gov NCT02839239. 

## 3. Results

**Figure 1:**
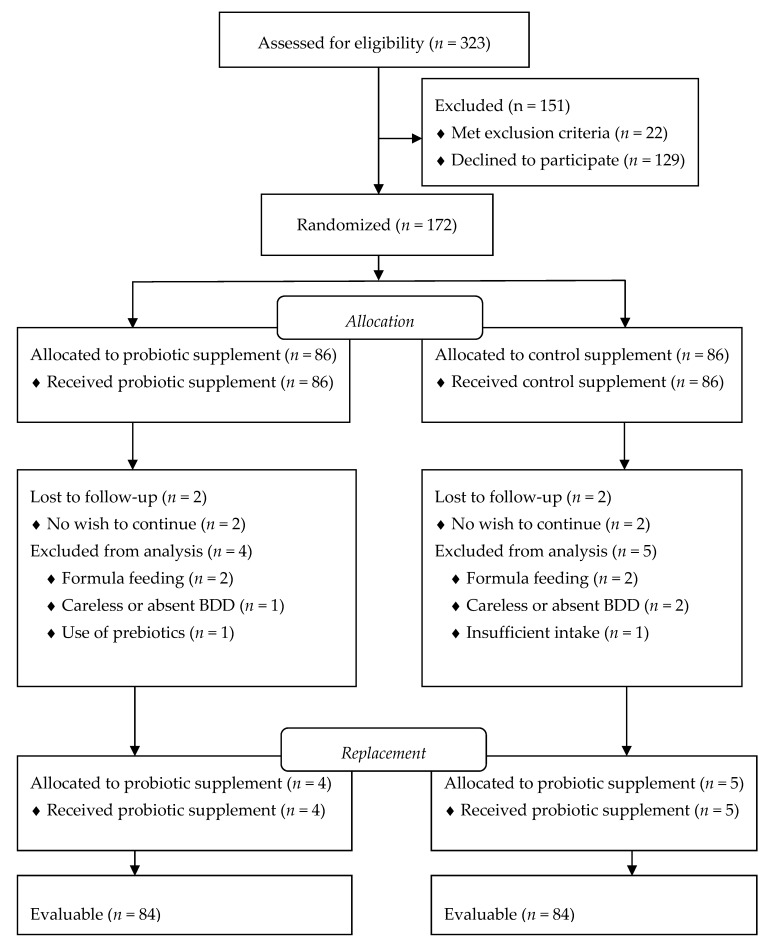
The Consolidated Standards of Reporting Trials flow diagram of the study participants. BDD: Baby`s Day Diary.

Screening and enrollment of infants occurred continuously from October 2016 to February 2018. Of 323 initially screened infants, 22 met exclusion criteria (formula feeding, present intake of probiotics, current maternal smoking, failure to thrive), and 129 declined to participate because of the investigational nature of the trial (Figure 1). During continuous evaluation of data, nine infants who finished the trial, were excluded from the analysis due to carelessly drawn BDDs, or failure to return BDD. One hundred seventy-two infants were randomized and evenly allocated into probiotic or control supplementation groups (86 infants/group). During the study, four infants were lost to follow-up with no further wish to participate. Four infants started formula feeding, three infants had carelessly recorded or absent BDD, one infant in the probiotic group started prebiotics, and one infant in the control group had poor compliance with intake of TDS These infants were excluded from the analysis and replaced with newly enrolled infants to secure pre-determined sample size. Finally, data from 168 infants were evaluable for study purposes.

### 3.1. Baseline Description of Study Participants

Probiotic and control groups were comparable on major demographic and medical data (Table 1).

A small percent of mothers and infants used probiotics and antibiotics in the past. No statistical difference was found between the groups, except for the duration of mother use of antibiotics and duration of infant use of probiotics, which were both longer in the control group. All probiotic and antibiotic treatments were stopped seven days before enrolment of the infant in the study. The most common past medical condition was hypoxic ischemic encephalopathy. In all cases, the diagnosis was made 1–3 days after delivery. The disease had mild severity and resolved by the time of enrollment. Functional jaundice was observed in approximately 5% (probiotic group) to 10% (control group) of newborns with no significant difference between the groups. Cephalo-hematoma was observed in one newborn in the probiotic and two newborns in the control group. At the time of inclusion, no finding of neurological consequences for the condition was found. Hemolytic disease, arthrogryposis, and lymphadenopathy were all documented in the probiotic group. Diaper dermatitis was observed in two infants in the probiotic group and one infant in the control group. None of the past medical conditions were moderate or severe, or was clinically active at time of enrollment, except for arthrogryposis that represents life-long congenital pathology of extremities.

It was found that around half of the sample size had a history of regurgitation (Table 2).

More than one third of infants suffered from constipation, and fewer infants were observed to have a vomiting-like episode, diarrhea or skin rash. There was no significant difference in prevalence of these conditions between the groups before the study enrollment, and none of these conditions had criteria sufficient for diagnosis. They were commonly described as occasional events, not clinically meaningful, and did not influence the condition of the infant. In all cases these infants were included in the study, and per investigator judgment, an occasional and mild episode of these conditions could not serve a confounder. 

One forth to one third of infants received treatments with claims for infant colic (Table 3). 

One fifth to one fourth of infants tried simethicone or dimethicone drops with no effects. Other treatments included probiotic dietary supplements, homeopathic drugs or fennel contained tea. All treatments were considered as ineffective by parents and finished at least seven days before entry in the study and were assumed not confounders for study outcomes.

### 3.2. Compliance

Generally, families complied well with the administration of the TDS and keeping the study diaries. During phone calls three families (3.6%) in the probiotic group reported one or more doses missed with a reference that this was due to forgetting and heavy daily routine. Two families (2.4%) in the control group reported doses missed for the same reasons. There was no statically significant difference in the number of infants who missed doses (*p* = 0.649). According to 28-DSD, of the total 4704 doses to be taken in one group (84 infants), only 11 doses (0.2%) were missed in the probiotic and 13 doses (0.3%)—in the control group (*p* = 0.331).

During phone calls, five families in the probiotic group and eight families in the control group reported significant troubles with recording in the BDD. In five cases in the probiotic group and four cases in the control group, the reason was difficulty in drawing graphical pictures. One family reported “difficulty to comprehend” on how to record in the BDD. Review and discussion of BDD in these families found consistent records.

### 3.3. Mother’s Diet

All infants in the probiotic and control were breast fed during the entire study period. Cumulative groups daily intake by mother of foods that suspected to produce IC revealed no statistical difference between the groups (Table 4).

The most common food used in both groups was cow’s milk that was consumed for approximately 40% of the study period. Approximately every 5th day mothers ate hen eggs, and approximately every 10th day chocolate and nuts. 

### 3.4. Outcomes

**Figure 2:**
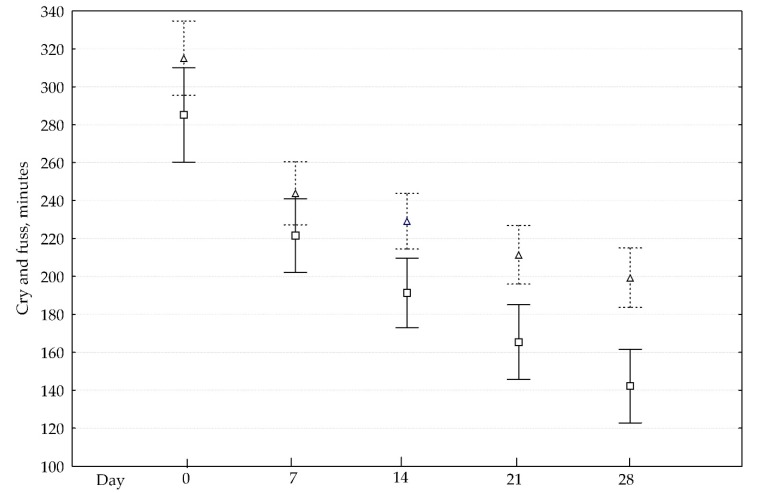
Cry and fuss time over study period. Data present as means (box mark – active; triangle mark - control group) and 2 standard errors (solid whiskers – active; dash whickers - control group).

There was no difference between the probiotic and control groups at baseline on the duration of fuss and cry (Figure 2).

After day 7, the probiotic group started demonstrating a faster reduction in cry and fuss time than the control group (upper boxes). Statistically significant results were found on days 14, 21, and 28 (*p* < 0.05). Mean change (SD) in cry/fuss time from day 0 through day 28 was −163 (99) minutes in the probiotic and −116 (94) minutes in the control group (*p* = 0.019). Success in controlling IC was more evident in the probiotic exposure group. Twenty-five percent reduction in cry/fuss times in the probiotic group was on day 8.2 (4.9) and day 9.6 (4.8) in the control group (*p* = 0.063). Fifty percent reduction in cry/fuss times in the probiotic group occurred after 8.9 (6.1) day and after 11.8 (7.4) day in the control group (*p* = 0.006).

**Figure 3:**
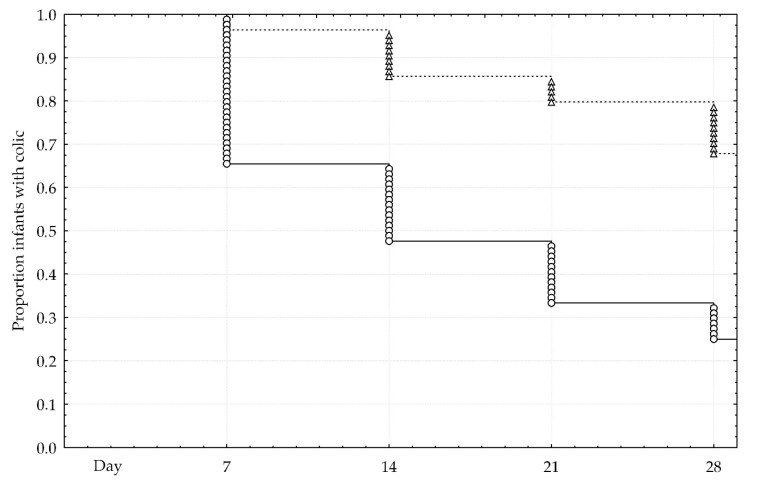
Recovery from colic. Solid line and round marks denote active group. Dash line and triangle marks denote control group.

Recovery from IC was illustrated in Kaplan-Meier analysis (Figure 3). Twenty-one infant (25%) in the probiotic group and 57 infants (68%) in the control group continued to have IC (*p* < 0.001). A significant difference between the groups was confirmed in Cox’s F-test. 

Mean (SD) sleep duration did not change due to study intervention. On enrollment it was 770 (87) vs. 773 (112) minutes (*p* = 0.847); on day 28th—795 (104) vs. 805 (82) minutes (*p* = 0.489) in the probiotic and control group, respectively.

Both groups showed marginal changes in EPDS. On enrollment, EPDS was 6.2 (3.3) vs. 6.0 (2.5) (*p* = 0.603); on day 28th—5.1 (1.8) vs. 5.4 (2.8) (*p* = 0.391) in the probiotic and control group, respectively. Mean EPDS changed −1.0 (2.9) in the probiotic and −0.4 (2.9) in the control group (*p* = 0.247) over the duration of the study period. 

On follow-up calls, parents in the probiotic group reported better course of IC than parents in the control group (Table 5). 

Phone call 1 elicited a greater number of infants with “better” colic in the probiotic group and fewer infants with “no changes” colic on day 12th of the study. Two infants from the probiotic group and four infants from the control group had worsening colic, however, the difference was not statistically significant. After phone call 2, the number of infants with a better condition was still greater, as well as fewer cases without changes. Three infants from the probiotic group and one infant from the control group had worsening colic, and the difference was also not statistically significant.

### 3.5. Intolerance Events

As reported by investigators, both probiotic and control interventions were tolerated well (Table 6).

The most common intolerance in the control group was the intensification of cry, followed by diarrhea and regurgitation. In the probiotic group these events were evenly presented by one infant per event, except for constipation. Eleven total intolerance events were observed in the control group compared to three total events in the probiotic group (*p* = 0.027).

## 4. Discussion

Study participants were between the ages of 30 to 60 days with equal representation of male and female gender. The age at entry was comparable with previous studies and averaged the age when the manifestation of colic was the most severe [29]. Earlier age at entry might theoretically underestimate the role of any intervention as infant cry/fuss, as well as cry/fuss in colic normally intensifies from the 4th to the 6th week of life, remaining relatively stable for the next two weeks. If infant cry/colic includes congenital/inherited component, infants entering the study before two weeks or after eight weeks might not respond to probiotic treatment. This might explain why studies with less or greater mean/median age failed to show probiotic effect [13,16]. 

In this study we showed that the combination of *L. rhamnosus* 19070-2 and *L. reuteri* 12246 was effective in alleviation of colic in breast fed infants. Our results are consistent with the recent meta-analysis of four randomized trials that differentiated outcomes of probiotic intervention in infants with different types of feeding. Generally, breast-fed infants receiving probiotics had a mean change in cry and/or fuss time from day 0 to day 21 of −156.0 min compared to −101.9 min in the control group (*p* < 0.05) [18]. In our study, the mean change was −163 and −116 min, respectively (*p* = 0.019). Lack of standard deviation figures in the mentioned study did not allow for study comparisons however trends were similar. Difference between the probiotic and control groups was −47 min (−163 − 116 = −47 min) with −40 min difference was planned during calculation of the sample size. The real time difference outnumbered the planned one, and this secured statistical power of the data obtained. 

In the meta-analysis data, the effect of lactobacilli assumed statistical significance from day 7, while in our research the difference was evident from day 14 [18]. This discrepancy at least partly can be explained by different approaches to collect primary data. In the referenced trials, only two used validated BDD on fixed days [13,15], while two others used non-standard scales with no indication on when these scales were used [12,17]. Even in the case of using a validated instrument, one may encounter a variability of data caused by the number of measurements. In a sample of 20 mothers, it was found that adequate monitoring of baby’s activity required at least a 3-day period [30]. As per correlation coefficient, the reliability of measurement of cry increased 0.329, 0.546, 0.989 with the incremental number of days (1, 2, or 3, respectively) the activity was recorded [30]. In the latter trial, mothers kept diaries only once for three days, while multiple measurements were planned in our study. To improve compliance, we requested parents to fill in the BDD one day before enrollment, and, thereafter, on pairs of consecutive days 6, 7, 20, 21, 27, 28. The double-day approach was also used in the study by Sung et al. to reduce daily variability of cry and fuss time [18].

Treatment success, defined as a 50% reduction of colic manifestation, was statistically significant in the breast-fed infants that received lactobacilli [18]. We followed a different methodology for the assessment of treatment success: (1) We calculated a mean time (days) when 25% or 50% reduction in cry and fuss was achieved, while other researchers assessed percent of infants experienced 50% reduction of cry/fuss at the end of intervention period; (2) We calculated 25% or 50% relative reduction in presentation from baseline 180 min, which is a cut-off for the diagnosis. As a result, 50% relative reduction of cry and fuss time was observed almost three days earlier in the probiotic group. In Kaplan-Meier analysis we found significantly faster total recovery from colic in the probiotic group as well. As with previous studies, it was not possible to determine the precise time to treatment success, as measurements were made weekly and not daily.

The effect of lactobacilli was documented at a daily dose significantly lower than one advocated by some regulatory authorities for probiotics [31]. In our study, the daily dose was 0.25 × 10^8^ CFU, while the minimum recommended number had been 1 × 10^9^ viable cells per day [I]. The rationale for the higher dose of probiotic in this statement was largely based on the results of studies in adults that cannot be directly translated for young infants. In IC, other investigators reported the positive effects of probiotics at daily doses of 10^8^ CFU [12,15,17,27]. Conversely, there had been a report that a dose as high as 10^9^ CFU/day did not exert the effect in IC [16], suggesting a lack of consensus on the dose.

Sleep duration, as a tertiary outcome measure, did not change in the study and was within normal limits for age [32]. Three of the present probiotic intervention studies did not report sleep duration as an outcome measure [12,15,17]. One study found no changes during probiotic or control feedings [13]. This could be due to extreme biological importance and dominance of sleep over other infant activities. It might be for the same reason that the feeding duration was not changed significantly as well. 

The role of breast-feeding modality on the positive effect of lactobacilli in infant colic is not understood. The situation is aggravated with the fact that breast-feeding itself did not affect the occurrence of colic [33,34] despite the fact it serves excellent nutrition medium for bifido- and lactobacilli [35,36]. The study performed by the Medical University of Vienna (Austria) also found a positive effect of probiotics on prevention of necrotizing colitis in the group of breast-fed infants [37]. Authors postulated that the effect can be explained by the presence of unique oligo-saccharides that promote optimal growth of lactobacilli, but direct studies comparing the potential of native human milk and herbal oligo-saccharides are not available. An alternative explanation of the lack of probiotic effect in formula fed infants may be due to a less “comfortable” physical and/or chemical environment of freeze-dried milk reconstituted in water for lactobacilli, however, direct data is also lacking.

The strength of our study was the randomized double blind controlled prospective parallel group design and the use of validated EPDS for measurement of maternal depression, and BDD for monitoring infant cry and fuss. The BDD tool was checked across sound records and found a reliable method for observation of infant cry and fuss [24]. Despite, there was not linguistic adaptation and validation of BDD, we believed that the translation of simple nouns, or combination of few words with clear meaning, could not significantly affect primary outcome measure. Linguistic validation of EPDS was available in the local language before the study started [26]. We retained almost all families primarily included in the trial, with phone calls and SMS notification messages. This gave an opportunity for evaluation and reassurance of the product storage, use of TDS, controlling for intolerance events, and timely filling the BDD. 

The study limitation was the inclusion of infants that were treated with antibiotics or/and received probiotics before inclusion in the study. The number of such infants was small (5 and 13 cases in the probiotic and control group, respectively), and all these interventions were finished more than seven days before study enrollment. The effect of this was known neither in our study nor in the referenced literature. Any history of antibiotic or probiotic use served exclusion criteria in the Canadian study [15]. In two studies, infants, who were treated with antibiotics or probiotics on the week before study enrollment, were excluded [12,17], while in another one only current intake of probiotics and antibiotics was an exclusion criterion [13]. No data was available for infants who received these treatments before the week of enrollment in any study, except for the study of Australian group, which reported that 22 infants took any probiotic before entry to the study [13]. 

The weakness of the study was the use of not validated 28-day study diary. The diary contained questions about maternal diet and possible gastro-intestinal upset symptoms in the infant. It was not known how much this truly reflected real life events. 

None of the infants received any treatments for colic during the study period. However, prior to the study more than 30% of infants received any type of treatment of colic that included simethicone or a similar drug, lactase, herbal medicine or homeopathic formulation. No consistent results were obtained for the efficacy of all the listed interventions except for fennel tea [7]. Only one randomized study successfully compared a herbal tea containing fennel with some other herbs with placebo. After one week of treatment, 57% of the infants in the active and 26% of the infants in the placebo group got rid of colic [38]. In our study, we had only one infant in the probiotic group who received a tea with fennel with no effect, and who stopped this treatment earlier than seven days before enrollment. 

Another limitation was due to the inclusion of infants with hypoxic-ischemic encephalopathy, whose brain injure may obscure colic or otherwise interfere with cry and fuss patterns in infants. Particularly, these infants may present transient behavioral abnormalities, such as poor feeding, irritability, or excessive crying or sleepiness in an alternating pattern. However, we included only infants with a mild course of the pathology, which recovers within a few days without consequences. [39]. Potentially, infants with cephalohematoma could influence the study results, as it sometimes presents with behavioral changes, such as increased sleepiness, and increased crying [40]. However, we included infants, who were fully asymptomatic, and at the discretion of investigator did not have any symptoms linked to this condition before enrollment. The criterion for this was symptom free interval before colic commenced. Other background health conditions as being all mild or producing no constitutional symptoms could not confound the study results.

Notably, that in this study we assessed pre-existed functional conditions/episodes to avoid labelling them as intolerance events. Indeed, regurgitation was seen in 55% and 63% of all infants before enrollment the study, in the probiotic and control groups, respectively. According to previous estimates, the infant may have functional regurgitation in up to 67% that meet our data [41]. As per the Rome criteria prevalence of functional constipation was 12.1%, vomiting 1.7%, and diarrhea 2.4% vs. in our study rates for constipation 31%/35%, vomiting 11%/12%, and diarrhea 21%/19% in the probiotic/control groups, respectively. The difference in rates of these conditions could occur due to different definitions of the cases. We defined the conditions once only one episode was seen, while according to Rome criteria, these conditions should last more than four weeks and occur more frequently than one time for the past period [42]. These functional conditions were similar in rates at baseline. However, analysis of total symptom load showed fewer days with regurgitation, constipation, or rashes in the probiotic group. Indrio F. et al., demonstrated that infants with functional regurgitation benefited from the use of lactobacilli [43]. In the probiotic group infants had fewer episodes of regurgitation that correlated with an increase of the gastric emptying rate and reduction of median fasting antral area. Later the same researchers reported more frequent stools in infants with functional constipation [44]. There were no identifiable reports about the efficacy of probiotics in pediatric functional diarrhea. Studies in adults with irritable bowel syndrome and diarrhea showed a decrease in stool frequency, but it is not known if this finding can be extrapolated for infants [45,46]. 

The significance of our findings is difficult to assess, as the frequency of gastro-intestinal symptoms was not pre-screened. It remains unknown if this was due to probiotic effect, or pre-study features of infants in two groups.

Finally, our study design did not permit ascribing positive effect of *L. reuteri* 12246, *L. rhamnosus* 19070-2, and FOS mixture to its separate constituents or their combinations. Most of previous studies explored a role of *L. reuteri* [12,15,17,27], *L. rhamnosus* [16], and FOS separately [47]. While *L. reuteri*-based showed consistent beneficial effects in breastfed infants, the study with *L. rhamnosus* showed no effect in nonhomogeneous group of breast- and formula-fed infants. Our infants were all exclusively breastfed, so the role of *L. rhamnosus* is difficult to discuss. Reduction in the manifestation of IC in the study of infants fed with FOS fortified formula also prevents from extrapolation of beneficial results obtained [47]. In this study, we calculated that one-month old infants received approximately 576 mg of FOS per day, assuming concentration of FOS of 0.8 g/100 mL and the normal daily volume of formula, of 720 mL. In our study amount of FOS was 3.33 mg, more than 170 times less of that in the mentioned study. However, we cannot exclude that FOS played a role in the propagation of lactobacilli in an infant’s intestines [48].

## 5. Conclusions

Our findings confirm that oral use of a mixture of *L. rhamnosus* 19070-2 and *L. reuteri* 12246 decreases cry and fuss time, and provides useful dietary support in exclusively breastfed infants with colic.

## Figures and Tables

**Table 1:** Baseline characteristics of study participants.

	Probiotic Group (*n* = 84)	Control Group (*n* = 84)	*p*-Value
Age, days, mean (SD)	44 (15)	45 (15)	0.666 ^1^
Gender male, *n* (%)	40 (48)	44 (52)	0.605 ^2^
Respiratory rate, mean (SD)	34 (3)	34 (3)	1.000
Heart rate, mean (SD)	132 (9)	132 (8)	1.000
Body weight, g, mean (SD)	4530 (637)	4581 (581)	0.588
Body length, cm, mean (SD)	55.9 (2.9)	56.6 (2.9)	0.119
Postnatal characteristics			
Weight at birth, g, mean (SD)	3427 (288)	3465 (278)	0.386
Length at birth, cm, mean (SD)	52.0 (2.0)	52.3 (1.9)	0.320
Head circumference, cm, mean (SD)	35.1 (1.7)	35.0 (1.5)	0.687
Gestation age, weeks, mean (SD)	39.4 (1.2)	39.5 (1.2)	0.589
Apgar score at 5 min., mean (SD)	8.3 (1.0)	8.2 (1.0)	0.518
Cesarean section, *n* (%)	7 (8.3)	9 (10.7)	0.597
Use of antibiotics and probiotics			
Use of antibiotic by mother, *n* (%)	7 (8.3)	8 (9.5)	0.785
Use of antibiotic by mother, days, mean (SD)	5.3 (2.1)	6.0 (1.1)	0.008
Use of probiotics by mother, *n* (%)	6 (7.1)	7 (8.3)	0.771
Use of probiotics by mother, days, mean (SD)	10.8 (5.4)	10.0 (6.2)	0.374
Use of antibiotics by infant, *n* (%)	3 (3.6)	2 (2.4)	0.649
Use of antibiotics by infant, days, mean (SD)	5.7 (1.5)	5.0 (9.4)	0.501
Use of probiotics by infant, *n* (%)	6 (7.1)	7 (8.3)	0.771
Use of probiotics by infant, days, mean (SD)	9 (3.3)	13 (9.3)	0.003
Hypoxic-ischemic encephalopathy, *n* (%)	11 (13.1)	16 (19.0)	0.299
Diagnoses			
Functional jaundice, *n* (%)	4 (4.8)	9 (10.7)	0.154
Umbilical hernia, *n* (%)	3 (3.6)	2 (2.4)	0.649
Cephalo-hematoma, *n* (%)	1 (1.2)	2 (2.4)	0.559
Hemolytic disease of a newborn, *n* (%)	1 (1.2)	0 (0.0)	0.315
Diaper dermatitis, *n* (%)	2 (2.4)	1 (1.2)	0.559
Arthrogryposis, *n* (%)	1 (1.2)	0 (0.0)	0.315
Lymphadenopathy, *n* (%)	1 (1.2)	0 (0.0)	0.315
Fever of unknown origin, *n* (%)	3 (3.6)	2 (2.4)	0.649

^1^ Difference between the groups in the Student’s *t*-test for means. ^2^ Difference between the groups in the z-test for proportions. SD, standard deviation; GIT, gastro-intestinal tract.

**Table 2:** Any gastrointestinal events or skin rashes before study enrollment.

	Probiotic Group (*n* = 84)	Control Group (*n* = 84)	*p*-Value ^1^
Regurgitation, *n* (%) ^2^	46 (55)	53 (63)	0.293
Vomiting, *n* (%)	9 (11)	10 (12)	0.839
Constipation, *n* (%)	26 (31)	29 (35)	0.582
Diarrhea, *n* (%)	18 (21)	16 (19)	0.746
Rash, *n* (%)	9 (11)	10 (12)	0.839

^1^ Difference between the groups in the z-test for proportions. ^2^ At least one episode of the listed events reported by parents was included in analysis.

**Table 3:** Prior treatment of colic.

	Probiotic Group (*n* = 84)	Control Group (*n* = 84)	*p*-Value ^1^
Simethicone, *n* (%)	17 (20.2)	22 (26.2)	0.358
Dimethicone, *n* (%)	3 (3.6)	3 (3.6)	1.000
Lactase, *n* (%)	9 (10.7)	7 (8.3)	0.597
*L. rhamnosus*, *n* (%)	2 (2.4)	2 (2.4)	1.000
*L. reuteri*, *n* (%)	3 (3.6)	3 (3.6)	1.000
Fennel tea, *n* (%)	1 (1.2)	0 (0.0)	0.315
Homeopathic, *n* (%)	0 (0.0)	1 (1.2)	0.315
*B. subtilis*, *n* (%)	1 (1.2)	2 (2.4)	0.559
Prior treatment of colic, *n* (%)	26 (31.0)	31 (36.7)	0.436

^1^ Difference between the groups in the z-test for proportions.

**Table 4:** Cumulative total-group days mother intake of foods suspicious to produce colic in breastfed infants.

	Probiotic Group (*n* = 84)	Control Group (*n* = 84)	*p*-Value ^2^
Cow’s milk, days, *n* (%)	973 (41.4) ^1^	937 (39.8)	0.264
Eggs, days, *n* (%)	499 (21.2)	474 (20.2)	0.397
Chocolate, days, *n* (%)	260 (11.1)	267 (11.4)	0.745
Nuts, days, *n* (%)	232 (9.9)	234 (9.9)	1.000

^1^ Data present as total days of intake of a specific food in the group (days of intake × number of mothers in the group) and percent days of all days of the study in the group (study duration (28) × size of the group (84) = 2352). ^2^ Difference between the groups in the z-test for proportions.

**Table 5:** Parental impression of colic during the study.

	Probiotic Group (*n* = 84)	Control Group (*n* = 84)	*p*-Value ^1^
Phone call 1			
Better, *n* (%)	58 (69.0)	39 (46.4)	0.035
Worse, *n* (%)	2 (2.4)	4 (4.8)	0.405
No changes, *n* (%)	24 (28.6)	41 (48.8)	0.008
Phone call 2			
Better, *n* (%)	78 (92.9)	66 (78.6)	0.008
Worse, *n* (%)	3 (3.6)	1 (1.2)	0.311
No changes, *n* (%)	3 (3.6)	17 (20.2)	0.001

Phone call 1 and 2 were made on 12 and 26 days of TDS intake. ^1^ Difference between the groups in the z-test for proportions.

**Table 6:** Intolerance events.

	Probiotic Group (*n* = 84)	Control Group (*n* = 84)	*p*-Value ^1^
Diarrhea, *n* (%)	1 (1.2)	3 (3.6)	0.311
Constipation, *n* (%)	0 (0.0)	1 (1.2)	0.315
Intense cry, *n* (%)	1 (1.2)	4 (4.8)	0.173
Regurgitation, *n* (%)	1 (1.2)	3 (3.6)	0.311
Intolerance events total, *n* (%)	3 (3.6)	11 (13.1)	0.027

^1^ Difference between the groups in the z-test for proportions.
